# In Utero and Lactational Exposure Study in Rats to Identify Replacements for Di(2-ethylhexyl) Phthalate

**DOI:** 10.1038/s41598-017-03979-0

**Published:** 2017-06-20

**Authors:** Thomas C. Nardelli, Océane Albert, Claudia Lalancette, Martine Culty, Barbara F. Hales, Bernard Robaire

**Affiliations:** 1 Department of Pharmacology and Therapeutics, McGill University, Montréal, Québec H3G 1Y6 Canada; 2 Department of Medicine, McGill University, Montréal, Québec H3G 1Y6 Canada; 3 Department of Obstetrics and Gynecology, McGill University, Montréal, Québec H3G 1Y6 Canada; 4 The Research Institute of the McGill University Health Centre, Montreal, Quebec, H4A 3J1 Canada

## Abstract

Di(2-ethylhexyl) phthalate (DEHP) and other phthalates are ubiquitous environmental contaminants with endocrine disrupting properties. Two novel plasticizers, 1,4 butanediol dibenzoate (BDB) and dioctyl succinate (DOS), have been proposed as potential replacements. Both have desirable properties as plasticizers and minimal *in vitro* biological effects. Herein, we present an *in utero* and lactational exposure study comparing DEHP with BDB, DOS, and 1,2-cyclohexane dicarboxylic acid diisononyl ester (DINCH), a commercial alternative. Timed-pregnant Sprague-Dawley rats were gavaged with vehicle or one of these chemicals at 30 or 300 mg/kg/day from gestational day 8 until postnatal day (PND) 21. The offspring were examined for effects on developmental and endocrine markers until PND 46. DEHP treatment (300 mg/kg) decreased heart weights in dams and induced a significant decrease in anogenital index and an increase in hemorrhagic testes and multinucleated gonocytes in PND 3 male pups. An increase in the incidence of hemorrhagic testes was also observed on PND 8 after exposure to DINCH (30 and 300 mg/kg). The only other effects observed were decreases in serum alanine transaminase and magnesium in BDB 30 exposed dams. These data suggest that both BDB and DOS are viable alternative plasticizers.

## Introduction

Phthalates are used as plastic emollients, matrices, solvents, and excipients in industrial applications^1^. Among these, di(2-ethylhexyl) phthalate (DEHP) is commonly used as an additive to provide flexibility to polyvinyl chloride (PVC). DEHP is found in a wide range of consumer products, including construction materials, toys, packaging films and sheets, medical tubing and blood storage bags^2, 3^. Since phthalates are not covalently bound to PVC, they leach from their matrices^4^ and become ubiquitous environmental contaminants^5^. Numerous studies have reported human exposure to these compounds^2, 6^. Despite their relatively rapid metabolism and excretion^7^, individuals are continuously exposed to these chemicals orally and, less commonly, by dermal, inhalational, and parenteral routes^8^. Early-life exposure to phthalates and their metabolites during pregnancy and early infancy also occurs as analytes are routinely detected in amniotic fluid^9^, umbilical cord blood^10^, and breast milk^11^.

Maternal exposure to phthalates during pregnancy is of concern based on data from numerous animal studies that have investigated effects on offspring^12, 13^. Decreased testosterone production^14, 15^, hemorrhagic testes^16^, multinucleated gonocytes in the seminiferous tubules^14, 16^, decreased anogenital indices^14, 16^, and nipple retention^16^ have been reported in the male offspring of dams exposed to DEHP during gestation. In females, phthalates have been shown to alter ovarian and oocyte development, folliculogenesis, the functionality of ovarian follicles and corpora lutea, and ovarian steroidogenesis (reviewed in ref. 13). Some studies have noted that the effects of DEHP and its metabolites extend beyond the reproductive system^17^. Gestational exposure has been reported to impair pancreatic β-cell function^18^ and lung maturation^19^, and to decrease blood pressure and locomotor activity^20^. In many cases, these effects have been observed many months after treatment was completed, suggesting that early exposures may have life-long effects even when the chemical or its metabolites are no longer detectable in biological samples.

Ethical restrictions, the combinatorial effects of other chemicals, and retrospective assessments of DEHP exposure^21^ have made human studies on the effects of phthalates particularly challenging. Despite these limitations, epidemiological studies indicate that phthalate exposure in humans is positively correlated with effects on genital development, such as decreases in anogenital index, decreases in semen quality, steroidogenic defects^22–24^, impaired cognitive and thyroid function, and respiratory problems (reviewed in ref. 25); many of these perturbations are associated with gestational exposure.

The growing body of evidence in support of the deleterious effects of phthalates has prompted legislation to protect consumers and limit daily exposure to these chemicals, particularly at an early age. The implementation of these measures has stimulated the search for safer replacements. Several commercial alternatives for DEHP have entered the market; 1,2-cyclohexane dicarboxylic acid diisononyl ester (DINCH) is one such alternative, but little is known about its potential toxicity. Previous studies have proposed 1,4 butanediol dibenzoate (BDB) and dioctyl succinate (DOS) as potential alternatives for DEHP; both compounds have desirable plasticizer properties, are relatively inexpensive to synthesize, and are biodegradable^26–28^. Both BDB and DOS have been shown to be devoid of toxicity in *ex vivo* organ cultures and *in vitro* functional and toxicogenomic assays^29, 30^. A 28-day acute toxicity study in post-pubescent male Sprague-Dawley rats demonstrated that neither of these plasticizers showed acute toxicity at doses of 15 or 150 mg/kg^31^.

The identification and characterization of toxicity in susceptible populations during critical windows of time is key to finding safe alternatives for phthalate plasticizers. The gestational and lactational windows of exposure represent a time when cellular identity and function are established via a myriad of signalling cascades, thereby making them vulnerable to xenobiotics^32^. The potential burden of early life exposure may be further amplified by the immature metabolic and excretion pathways of neonates^33^. These inherent vulnerabilities make fetuses and newborns an important population to examine when assessing the toxicity of a replacement compound.

Herein, we hypothesized that *in utero* and lactational exposure to three replacements for DEHP (BDB, DOS, and DINCH) would have fewer or no deleterious effects on the general health of offspring exposed during gestation and lactation, and would not have the endocrine disruptive effects that target the reproductive system and are characteristic of DEHP. The goal of these studies was to identify suitable alternative plasticizers that could be used for the manufacturing of PVC based plastics.

## Results

**Figure 1:**
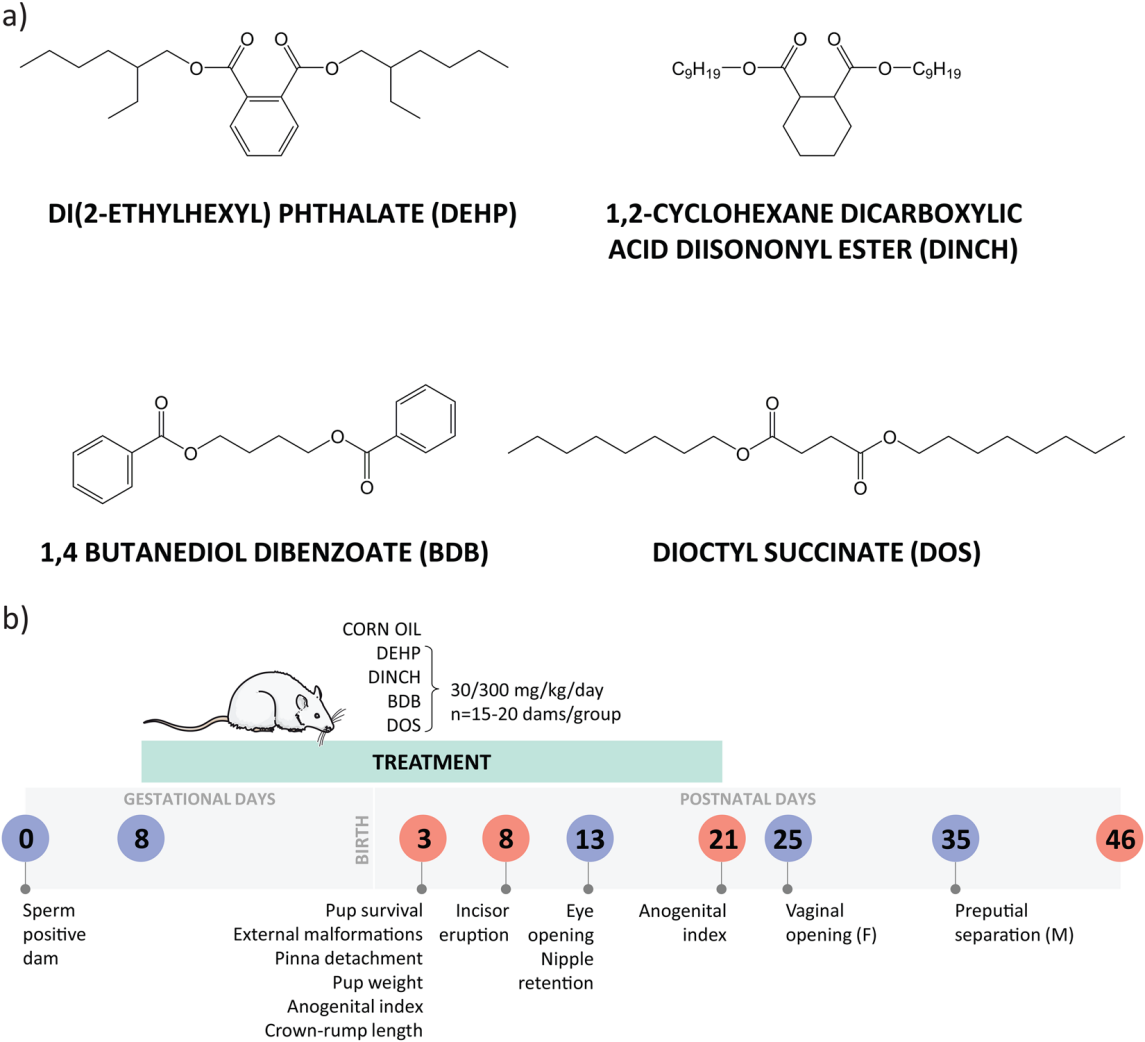
Chemical structures and experimental design. (**a**) Chemical structures of each plasticizer. DEHP is the current and most commonly used phthalate plasticizer. DINCH is a commercial alternative that is being marketed as a safer replacement. BDB and DOS are two alternatives that have been developed and tested, but are not commercially available for use. (**b**) Experimental schematic showing key endpoints. Timed pregnant dams were treated from GD8 until weaning with one of nine treatments (including control). Pups were observed for reproductive and developmental endpoints; on the specified days one pup from each litter was selected at random for necropsy. The rat illustration (http://www.servier.com/slidekit/?item=13) is from Servier Medical Arts Powerpoint Image Bank, Creative Commons Attribution 3.0 Unported License (https://creativecommons.org/licenses/by/3.0/).

The effects of DINCH, BDB and DOS (Fig. 1a), three plasticizers that are currently in use or have potential as alternatives to DEHP, were investigated in timed-pregnant Sprague Dawley rats exposed during gestation and lactation (Fig. 1b).

### Maternal Health

**Figure 2:**
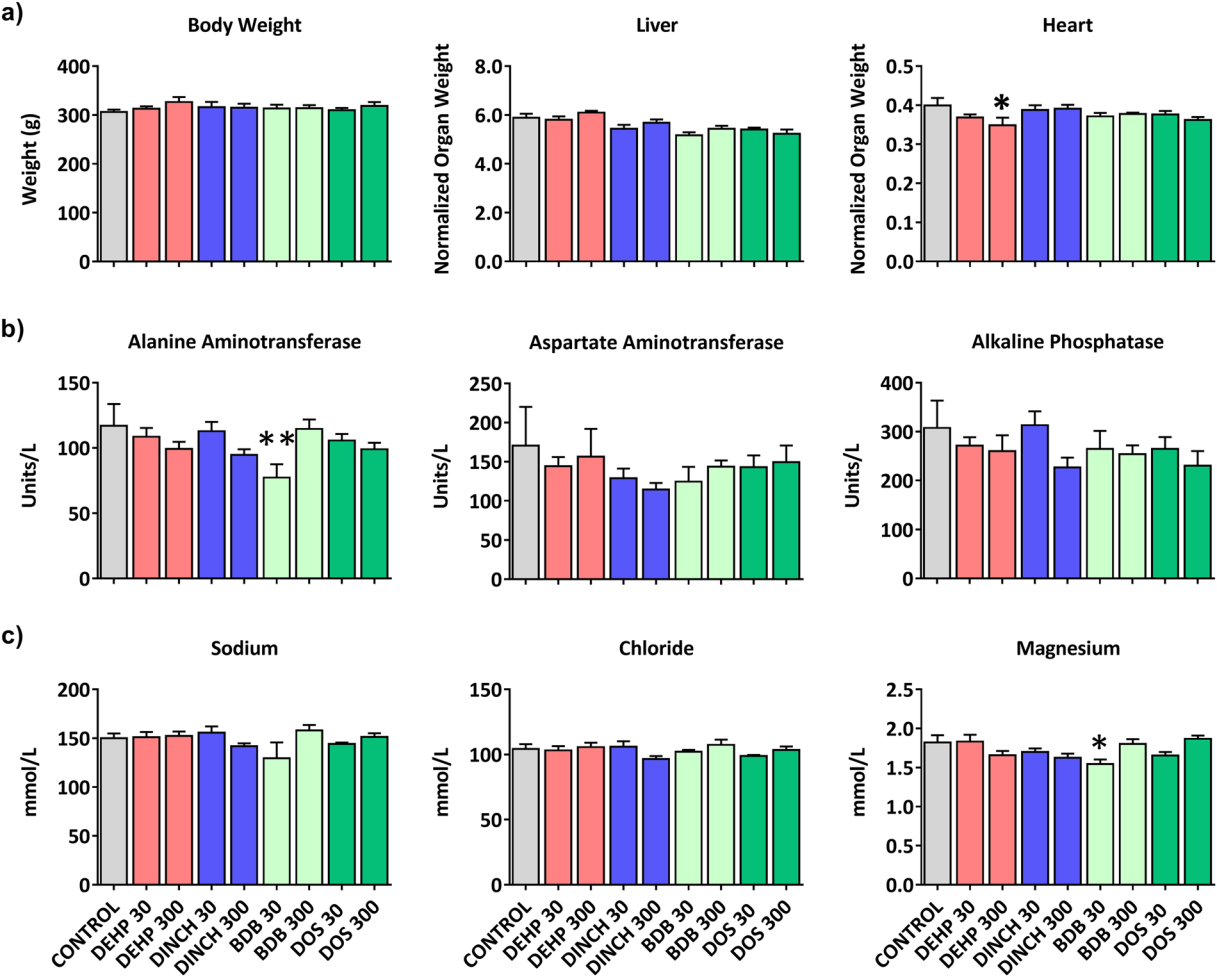
Organ weight and serum parameters of dams at weaning. (**a**) Animal weight and organ weights normalized to body weight are reported. n = 11–16. Bars represent means ± SEM. One-way ANOVA, post-hoc Dunnett’s, *p ≤ 0.05. (**b**) Selection of serum biomarkers related to liver function (alanine aminotransferase, aspartate aminotransferase, and alkaline phosphatase) function. (**c**) Electrolytes measured from serum samples. n = 10. Bars represent means ± SEM. One-way ANOVA, post-hoc Dunnett’s, *p ≤ 0.05, **≤0.01.

The overall health of dams was assessed during the course of treatment and at necropsy on postnatal day (PND) 21. Five dams died spontaneously over the course of the study but these animals were not restricted to any particular treatment group; one rat was from the control group, one from DINCH 30 (30 mg/kg/day), two from DINCH 300 (300 mg/kg/day), and one from the BDB 30 (30 mg/kg/day) treatment group. Necropsies indicated that the cause of death for one rat (BDB 30) may have been gavage error but did not reveal any cause of death for the others. The remaining dams all appeared healthy, gained weight and did not show physical symptoms of distress. At the time of necropsy there was no significant effect of treatment on body weight (Fig. 2a). While dam liver, kidney, lung, spleen, ovary, and uterus weights were unaffected by treatment (Fig. 2a and Supplemental Table S1); a decrease in heart weight was observed in the DEHP 300 group (Fig. 2a).

Serum was collected to identify changes in biomarkers associated with pathogenesis. Among the serum biomarkers analyzed, only alanine transaminase and magnesium were affected and only in the serum of BDB 30 dams (Fig. 2b,c and Supplemental Table S2). While an increase in alanine transaminase typically is associated with liver pathogenesis, a decrease in this enzyme is not considered to have untoward effects. The small (~10%) but significant decrease in serum magnesium may represent mild kidney dysfunction. In either case, complementary markers of kidney and liver function were unchanged.

### Pregnancy Outcome

Several endpoints were used to determine whether exposure during pregnancy affected pregnancy outcome. Uteri from postpartum dams were collected and implantation scars within the uterine horns were counted to determine the number of implantation sites. The numbers of viable pups were counted on PND 3 and their sex was determined. There were no significant effects of treatment on post-implantation loss, pup survival, or sex ratio, suggesting that litter characteristics were not affected (Table 1).

**Table 1:** Experimental design and litter parameters.

Treatment Group	Treated Animals	Animals Included	Implantation Scars/Litter	Pups/Litter (PND 3)
CORN OIL	19	14	15.4 ± 0.8	12 ± 3.7
DEHP 30	17	14	14.7 ± 0.8	13 ± 3.4
DEHP 300	18	12	13.8 ± 1.1	11 ± 4.1
DINCH 30	19	11	13.4 ± 1.1	10 ± 4.7
DINCH 300	16	13	16.4 ± 0.4	13 ± 3.0
BDB 30	16	12	16.0 ± 0.5	13 ± 3.5
BDB 300	17	16	15.2 ± 0.4	13 ± 3.2
DOS 30	17	14	14.5 ± 0.9	13 ± 3.2
DOS 300	15	14	14.5 ± 1.3	13 ± 4.6

Dam and litter parameters: all animals were sperm positive on GD 0 and were assigned to a group on GD 8 when treatment began. Only litters with greater than six pups met the inclusion criteria for the study’s endpoints from GD 8–46. To ensure that the effect of small litters was not due to treatment, implantation scars were counted by transillumination of uteri dissected from dams at the time of weaning (PND 21). Pups that survived to PND 3 were also counted. There was no significant effect of treatment on litter size or pup survival. Values reported are the means ± SEM.

There were no effects of treatment on the numbers of external malformations observed on PND 3 in any treatment group, but one pup was missing a tail (DEHP 300) and two pups appeared to have hydrocephalus (DOS 30, DOS 300). Pup weights and crown-rump lengths on PND 3 were not affected by treatment (Supplemental data Table S3). Developmental markers, such as incisor eruption and pinnae detachment, were not affected by treatment. At PND 21 and 46, there were no effects of treatment on organ weights (liver, spleen, kidney, heart, lung, ovary, uterus, epididymis or testis; Supplemental Tables S4 and S5).

### Endocrine and Reproductive Effects on Female Offspring

**Figure 3:**
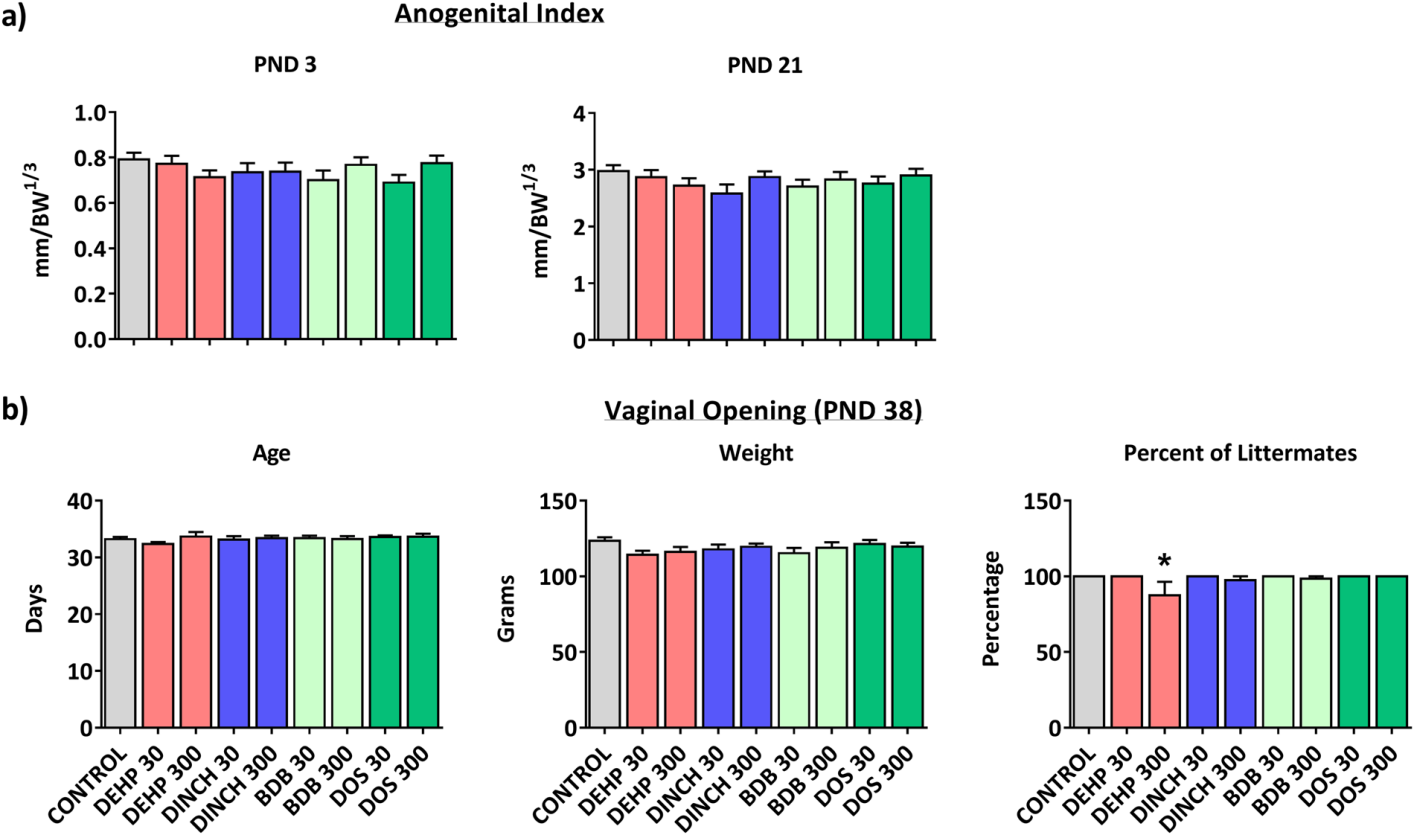
Female markers of endocrine function. (**a**) Anogenital index of female pups at PND 3 and 21. (**b**) Average age and weight at the time of vaginal opening. This panel also includes the average percent of littermates that completed vaginal opening by PND 38. Bars represent means ± SEM; n = 11–16. One-way ANOVA, post-hoc Dunnett’s, *p ≤ 0.05.

Functional markers of reproductive development and endocrine function were monitored in female pups. There were no significant effects of treatment on anogenital index (AGI) in female pups at PND 3 and 21 (Fig. 3a). Vaginal opening was monitored daily as a marker of pubertal onset. While the age and weight of vaginal opening averaged within a litter did not change, the litter index at PND 38 (defined as the mean percentage of pups in a litter with completed vaginal opening at PND 38) was significantly decreased in the DEHP 300 group (Fig. 3b).

**Figure 4:**
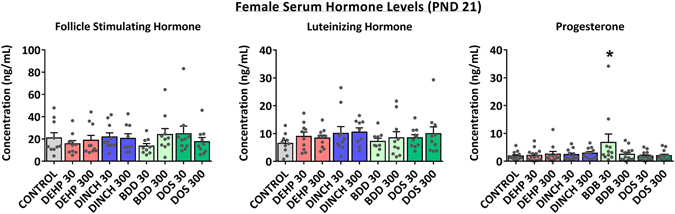
Serum hormone levels in female offspring at PND 21. Each grey point represents an animal selected at this time-point for necropsy. Bars represent means ± SEM; n = 10. One-way ANOVA, post-hoc Dunnett’s, *p ≤ 0.05.

Serum hormone levels were measured at PND 21 to determine whether exposure affected steroid hormone production. While LH and FSH concentrations were not affected by any treatment, there was a significant increase in progesterone following treatment with BDB 30 at PND 21; however, this observation should be interpreted with caution as two values that were included in the analysis differed greatly from the mean and thus may be considered as outliers (Fig. 4). Estradiol levels were below the detection limit of the assay on PND 21.

A quantitative PCR gene panel was used to investigate the potential impact of DEHP and the alternative plasticizers on the female reproductive system. The expression of steroidogenic enzymes remained unchanged in all treatment groups (Supplemental Figure S1). Furthermore, there were no significant changes in the expression of genes important for reproductive function (Supplemental Figure S2).

### Endocrine and Reproductive Effects on Male Offspring

**Figure 5:**
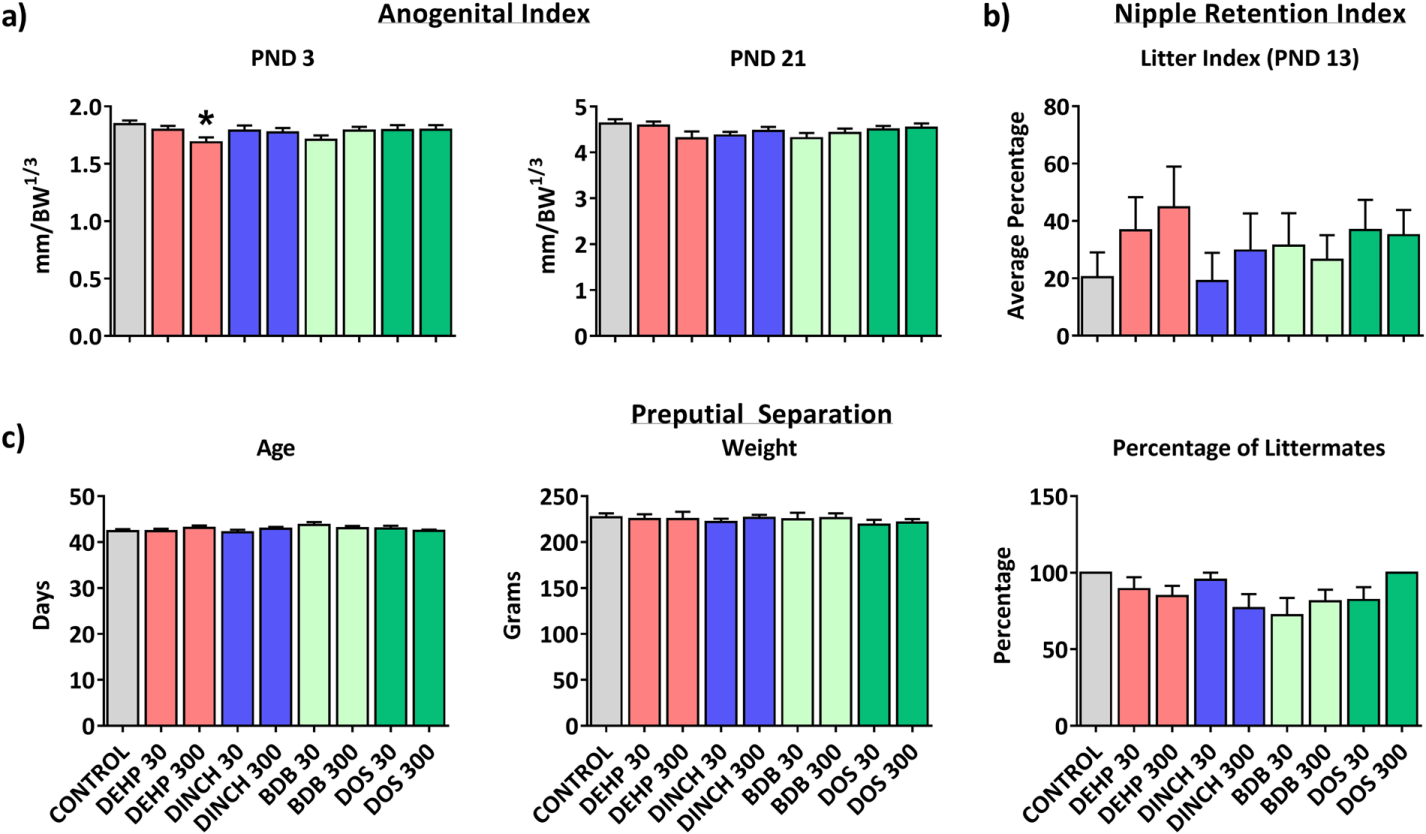
Markers of male endocrine function. (**a**) AGI at PND 3 in male pups. (**b**) Nipple retention in male pups at PND 13. (**c**) Average age and weight at the time of preputial separation. This panel also includes the average percent of littermates that completed preputial separation on or before PND 46. Bars represent means ± SEM; n = 11–16. One-way ANOVA, post-hoc Dunnett’s, *p ≤ 0.05.

Functional markers of reproductive development and androgen action were monitored in male pups. AGI was significantly decreased at PND 3 in the DEHP 300 mg/kg treatment group, but remained unchanged at PND 21 (Fig. 5a). The presence of retained nipples at PND 13, a marker of impaired androgen action, was not significantly altered (Fig. 5b). Preputial separation was also monitored as a marker of the onset of puberty (Fig. 5c). There was no significant change in preputial separation measured at PND 46.

**Figure 6:**
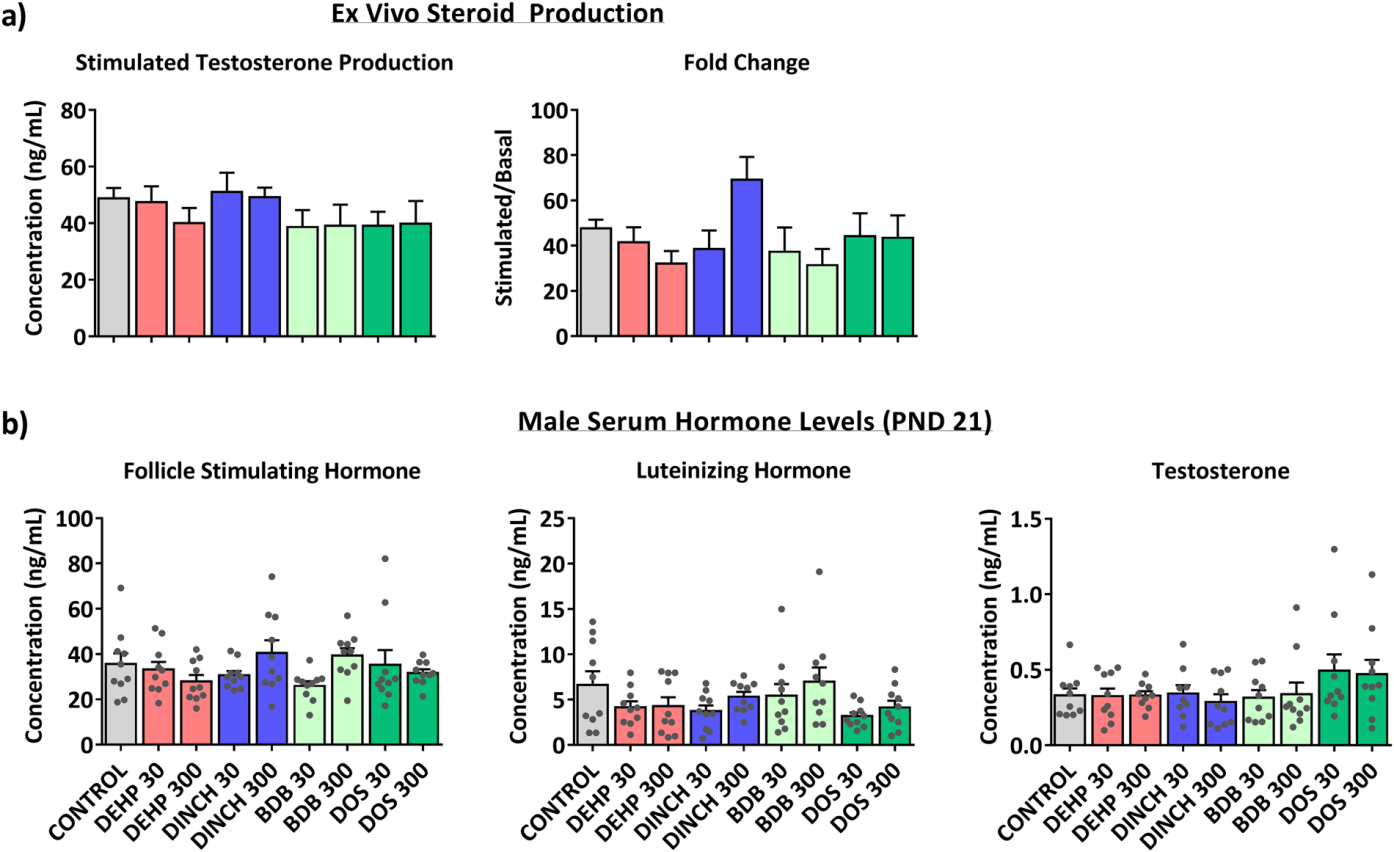
Assessment of steroidogenesis. (**a**) *Ex vivo* testosterone production assay. PND 3 testes from each animal were tested in both the basal and stimulated conditions. Fold changes (stimulated/basal) were determined for each animal and averaged across the treatment group. Bars represent means ± SEM; n = 7–11. One-way ANOVA, post-hoc Dunnett’s (**b**) Serum hormone levels in male pups at PND 21. Bars represent means ± SEM; n = 10. One-way ANOVA, post-hoc Dunnett’s. Grey points represent an individual animal.

As development of the perineum is dependent on androgen action, basal and hormone-stimulated testosterone production were measured in PND 3 testes using an *ex vivo* testicular culture system. There was no significant change in testosterone production under either stimulated or basal conditions (Fig. 6a). Each animal provided material for the basal and stimulated conditions, therefore average fold change per animal was also calculated, but was not significant. Serum was also collected from male offspring to determine whether there was an effect of treatment on steroidogenesis. There were no significant changes in serum FSH, LH, or testosterone at PND 21 (Fig. 6b) in any treatment group.

The potential impact of plasticizers on testicular gene expression was examined using a quantitative PCR gene panel. On PND 21 an increase in Hsd17b3 expression was observed in the BDB 30 group (Supplemental Figure S3). There were no significant effects on the expression of other steroidogenic enzymes or other genes of reproductive relevance (Supplemental Figure S4).

**Figure 7:**
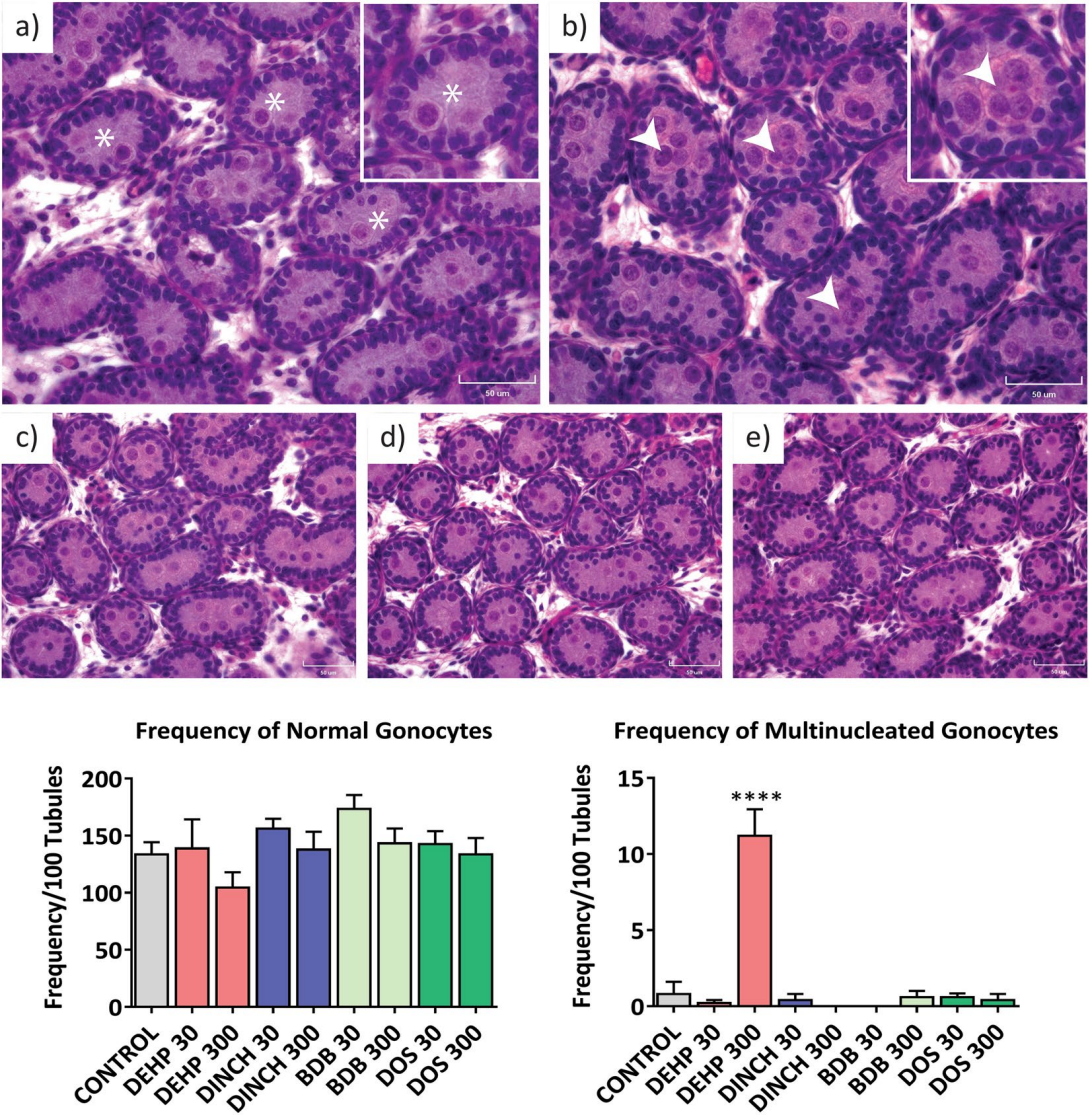
Histological examination of testes at PND 3. High quality photomicrographs of PND 3 testes following exposure to (**a**) corn oil, or 300 mg/kg: (**b**) DEHP, (**c**) DINCH, (**d**) BDB, and (**e**) DOS (**e**). Normal gonocytes are indicated with an asterisk; multinucleated gonocytes are indicated with white arrowheads. (**f**) Multinucleated and (**g**) normal gonocytes were quantified from 100 perpendicular cross sections of seminiferous tubules. Bars represent means ± SEM, n = 5. Scale bar represents 50 µm. One-way ANOVA, post-hoc Dunnett’s, ****p ≤ 0.0001.

**Figure 8:**
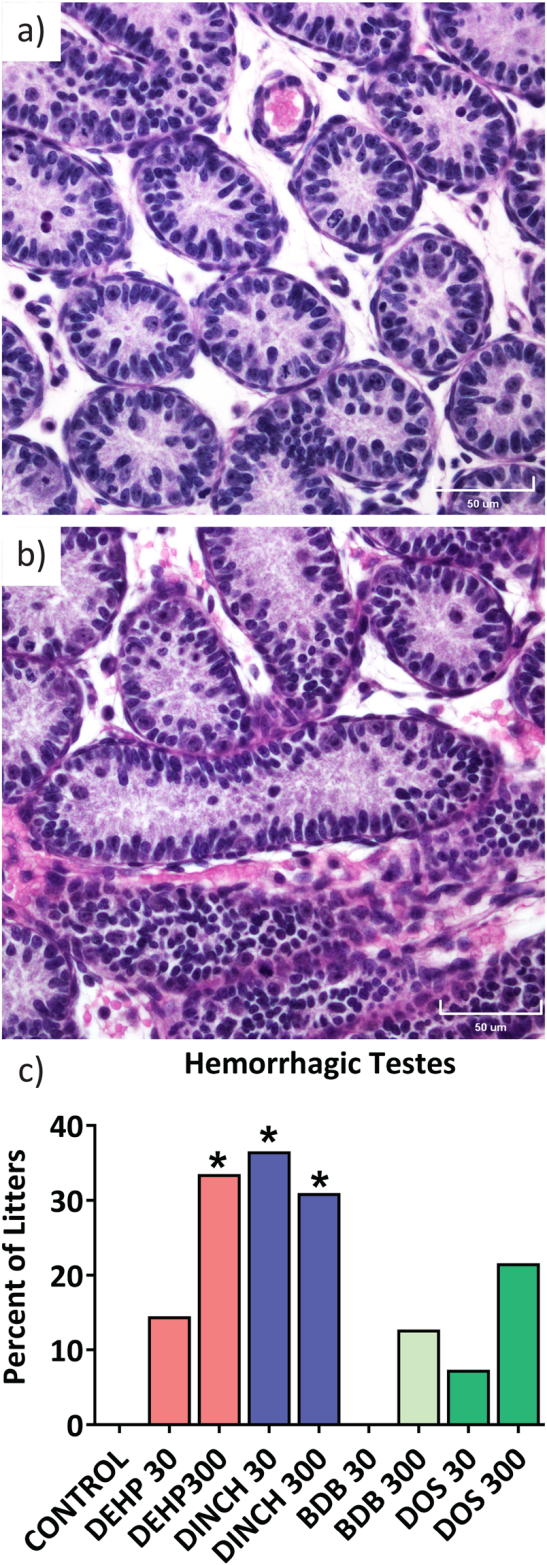
Histological analysis of testes at PND 8. High quality photomicrographs of PND 8 testes displaying (**a**) normal histology or (**b**) partial hemorrhage. The occurrence of testicular hemorrhage at PND 8 was quantified (**c**). Values are percent of litters with partial or full hemorrhage. Scale bar represents 50 µm. n = 11–16. Fisher’s exact test, *p ≤ 0.05.

We also investigated the effects of plasticizers on testicular histology. The presence of multinucleated gonocytes in seminiferous tubules has been associated with exposure to DEHP in several studies; this has been observed in the absence of measurable decreases in testicular testosterone^34^. Figure 7 shows representative histological sections from PND 3 testes from: (a) control; (b) DEHP 300, with an insert highlighting a multinucleated gonocyte; (c) DINCH 300; (d) BDB 300; and (e) DOS 300 offspring. The number of normal gonocytes was not affected by treatment (Fig. 7f). However, we observed a significant increase in the number of multinucleated gonocytes in the testes of offspring treated with 300 mg/kg/day of DEHP (14-fold compared to control; p ≤ 0.0001) (Fig. 7g). No increase in multinucleated gonocytes was observed in any of the other treatment groups. Hemorrhagic spots or patches were observed in testes on PND 3 and 8. Testes were considered hemorrhagic if they had red spots, patches, or were fully hemorrhagic upon dissection. A significant increase in the incidence of hemorrhagic testes was observed at PND 8 in the DEHP 300 treatment group (Fig. 8c). Interestingly, the DINCH 30 and DINCH 300 treatment groups also showed a significant increase in the incidence of hemorrhagic testes; this was not significant in other treatment groups. At PND21, the presence of hemorrhagic testis was not observed, and testicular histology did not show any major abnormalities in any treatment group (not shown).

## Discussion

Herein, we present an *in utero* and lactational exposure study comparing DEHP and 1,2-cyclohexane dicarboxylic acid diisononyl ester (DINCH) with two candidate alternative plasticizers, BDB and DOS, chosen on the basis of their plasticizing, leaching and biodegradability properties^26–28^ and minimal biological activities in immortalized cell lines derived from male reproductive tissues^29, 30^. We report that *in utero* and lactational exposure to BDB and DOS did not produce the endocrine disruptive phenotype classically described after exposure to DEHP. While DINCH had no effects on many of the endpoints associated with DEHP exposure, it did significantly increase the incidence of hemorrhagic testes in exposed offspring.

Treatment with 300 mg/kg/day DEHP significantly decreased the anogenital index and increased the number of multinucleated gonocytes in PND 3 male rat offspring; on PND 8, this exposure significantly increased the incidence of hemorrhagic testes. Our experimental model is clearly responsive to the effects of DEHP since we observed an effect on the anogenital index at a lower dose (300 mg/kg/day) than that used in a number of previous studies in the literature (750 mg/kg/day)^14, 16^. Previous studies reported a decrease in testosterone production after *in vitro* ^35, 36^ and *in utero* ^15^ exposure to DEHP or its monoester metabolite, MEHP. We did not note any significant alteration of testosterone production by *ex vivo* testicular explants, in serum testosterone levels, or in measures of biologically active testosterone such as seminal vesicle weights on PND 21 and 46 (Supplemental Tables 4 and 5). Differences between our study and previous studies may be due to the dose, window of exposure, and/or strain of rats used; all of these parameters are potential sources of discrepancy in the study of anti-androgenic compounds^12^. While profound effects on male reproductive function have been reported after treatment with DEHP at doses upwards of 750 mg/kg, it is also clear that deleterious effects on physiology that extend beyond the reproductive system have been observed after treatment with lower doses of DEHP and other phthalates^37, 38^. Exposures in this study started as early as GD8 to target a wide window during embryo and fetal development that includes a period of susceptibility during the development of multiple organ systems. Many studies characterizing the anti-androgenic effects of *in utero* phthalate exposures start treatment at GD14, a time that is critical for androgen action on gonadal differentiation^39, 40^. Finally, Sprague-Dawley rats were selected as an outbred model that is commonly used in toxicology studies. While there are differences in response to phthalates between rat strains, steroidogenesis in Sprague-Dawley rats is perturbed following DEHP exposure, resulting in abnormalities in androgen dependent organs^41^. These perturbations in steroidogenesis are similar to those observed in studies with human testicular explants^42^.

The effects of phthalates as endocrine disruptors in female animals have been largely unexplored. This is of particular concern as women at all ages are exposed to phthalates^9^. Here, DEHP (300 mg/kg/day) delayed the completion of vaginal opening, a marker of sexual maturation, in female offspring. Such a delay was described previously in Wistar rats after *in utero* and lactational exposure to DEHP^43^. These findings highlight the need for additional research on mechanisms by which phthalate plasticizers act as endocrine disruptors of female reproductive function.

Interestingly, DEHP also caused a decrease in heart weight in dams. The literature on phthalates and cardiac function is limited. Isolated cardiomyocytes treated with DEHP *in vitro* have been reported to have increased levels of reactive oxygen species from fatty acid metabolism, possibly resulting in an increase in the susceptibility of the heart to ischemic injury and ventricular dysfunction^44^. DEHP has also been reported to dysregulate electrical conduction and mechanical contraction in isolated neonatal cardiomyocytes by altering gene expression^45, 46^. These findings suggest that further work is needed to understand the impact of DEHP on cardiomyocytes and heart function.

We report a significant increase in the occurrence of testicular hemorrhage in male offspring at PND 8 after exposure to high dose DEHP and to both the low and high doses of DINCH. DINCH was first introduced to the European market in 2002, received final approval from the European Food Safety Authority in 2006, and has been marketed since as a safe phthalate replacement. Nevertheless, there is limited knowledge about its safety or potential adverse health effects although widespread exposure, as assessed by increasing urinary levels of DINCH metabolites, has been documented^47, 48^. Recent *in vitro* studies suggest that DINCH is a bioactive compound^29^, a potent metabolic disruptor^49^, and an endocrine disruptor^30^. The increase in hemorrhaging testes that we observed after DINCH exposure occurred at doses that were below or equal to the no observed adverse effect level (NOAEL) for parenteral exposure^50^. Together, these data suggest a need for a deeper exploration of the possible endocrine disruptive properties of DINCH.

Importantly, BDB and DOS, our candidate compounds, did not significantly increase the incidence of classically described endocrine disruptive phenotypes. Among the significant effects produced by our compounds, the decrease in alanine transaminase and magnesium in pregnant dams treated with 30 mg/kg/day BDB is an unusual finding. An increase in serum alanine transaminase is commonly used as a biomarker of liver damage^51^. The finding of a decrease in alanine transaminase, accompanied by a lack of significant change in other biomarkers of liver damage, is inconsistent with the possibility of liver toxicity. Hypomagnesemia may cause perturbations in most organ systems as magnesium is a key activator for enzymatic reactions involving phosphorus^52^. Magnesium homeostasis is regulated in the gastrointestinal tract, where it is absorbed, and in the kidneys, which ultimately determine whether it is eliminated or reabsorbed in the proximal tubules^52^. Again, this finding of a small but significant change in serum magnesium is difficult to interpret as there is no indication from other biomarkers or electrolytes that there is kidney impairment and the animals were otherwise healthy and gained weight. In both cases, further studies may be warranted.

There were negligible changes in gene expression at PND 21 in the testes or ovaries of offspring. Hsd17b3 was the only gene to be significantly upregulated in this study, following treatment with BDB 30 in male rats. While there are several isoforms of this enzyme, Hsd17b3 is expressed primarily in the testes and is a marker of adult Leydig cells^53^. Furthermore, this enzyme preferentially converts androstenedione to testosterone^53^. This change in Hsd17b3 gene expression was not associated with any effects on serum testosterone, *ex vivo* testosterone production, or androgen-sensitive markers, suggesting that testosterone biosynthesis is unaffected in this model.

With an annual economic burden estimated at $340 billion in the United States^54^ and more than €150 billion in Europe^55^, endocrine disruptors have become one of the most prominent public health issues in modern society. In previous studies we identified two novel candidate plasticizers, BDB and DOS, with desirable plasticizing properties and biodegradability, minimal leaching, and an absence of toxicity in several immortalized cell lines. Here we demonstrate that *in utero* and lactational exposure to these compounds produced fewer or no significant adverse effects compared to DEHP. Both plasticizers have been subject to more extensive screening than most new chemicals, thereby promoting the use of responsible replacements for future generations.

## Materials and Methods

### Chemicals and Reagents

Di-2-ethylhexyl phthalate (DEHP) was purchased from Sigma-Aldrich Corporation (CAS #117-81-7; Cat#80030, St. Louis, MO). 1,2-Cyclohexane dicarboxylic acid diisononyl ester (DINCH) (CAS # 474919-59-0 and 166412-78-8) was supplied by BASF Canada (Mississauga, ON). 1,4 butanediol dibenzoate (BDB) and dioctyl succinate (DOS) were synthesized as previously described^26, 28, 56^. Chemical structures are shown in Fig. 1a. The purity of BDB and DOS was determined to be 99% by NMR analysis (unpublished data). Chemicals were stored in a vacuum chamber with desiccant at room temperature until mixed with corn oil (Catalogue #C8267; Lot#MKBN5383V, Sigma-Aldrich).

### Animal Husbandry

All manipulations and terminal procedures were in accordance with protocol #7317, approved by the McGill University Animal Care Committee. This experiment was divided into three cohorts. Virgin female and proven-breeder male Sprague-Dawley rats were purchased from Charles River Laboratories (St-Constant, Quebec). Animals were kept on a 12-hour light/dark cycle and provided food and water *ad libitum*. Females in proestrus were placed in mating cages that contained two females and one male rat. Sperm positive females were placed in individual cages the next morning; this was considered gestational day (GD) 0. Animals were randomly assigned to vehicle (corn oil, Sigma-Aldrich), DEHP, DINCH, BDB, or DOS treatment groups on GD 8, when treatment began; each treatment was prepared daily. Animals were weighed and administered doses of 30 or 300 mg/kg by gavage. The lower dose (30 mg/kg) is representative of high human exposure to DEHP^57^ with an adjustment for interspecies metabolism^58^. The higher dose (300 mg/kg) was selected based on previous literature that reported deleterious reproductive outcomes following exposure to DEHP during gestation and lactation^37^. Thus, the treatment doses were chosen based on studies with DEHP; since DOS and BDB readily biodegrade and have slower leaching rates than DEHP, we expect that if these chemicals were released into the environment human exposures would be lower than for DEHP. Control animals were administered 1 ml of corn oil; this was the maximum volume administered for any treatment. Dams were treated daily, with the exception of post-natal day (PND) 0 when the litters were not disturbed. Treatment continued until weaning, which occurred on PND 21. Terminal and developmental endpoints are summarized in Fig. 1b.

### Inclusion Criteria

All animals that produced a litter were included in the study until PND 3 regardless of litter size. At PND 3, litters were culled to eight pups each. Litters with less than six pups at this time were excluded from future endpoints (Table 1).

### Terminal Endpoints

Pups were euthanized at PND 3 and PND 8 by inducing hypothermia followed by decapitation. Dams and pups from PND 21 onwards were euthanized by CO_2_ asphyxiation followed by cardiac puncture. Organs were weighed and collected for histology and RNA isolation.

### Developmental Endpoints

#### Anogenital Index (AGI)

Average pup body weight (BW) over 10 seconds was determined using a NewClassic MF balance (Mettler Toledo, Mississauga, ON). The anogenital distance (AGD) was measured using a pair of Vernier digital calipers, as previously described^59^. AGI is defined as AGD/(BW)^1/3^. AGI was calculated for each pup; the litter was considered as one unit and litter data were averaged for each treatment group.

#### External Malformations and Developmental Endpoints

At PND 3, each pup was closely examined for external malformations. Examiners were blinded as to the treatment group. The inspection included the head, digits, body, and tail. Several hallmarks of development were recorded over the study period; these included crown-rump length, pinnae detachment (PND 3), incisor eruption (PND 8), eye opening (PND 13), nipple retention (PND 13), vaginal opening (PND 25–43) and preputial separation (PND 35–46), as previously described^60^. Litter index represents the average percentage of littermates that completed either vaginal opening or preputial separation by PND 38 or 47 for females and males, respectively.

### Tissue Processing

#### Histology

Organs were collected and fixed in Modified Davidson’s fixative (30% of a 37–40% solution of formaldehyde, 15% ethanol, 5% glacial acetic acid, and 50% distilled water) for 24 hours at room temperature. Larger organs were cut into smaller pieces for better fixation. The next day, the fixative was removed and replaced with 70% ethanol for storage at 4 °C.

#### RNA Isolation

Organs were collected, placed in cryogenic tubes, and immediately immersed in liquid nitrogen. Entire frozen testes from PND 21 rats were mechanically disrupted using a Polytron PT 10–35 GT homogenizer (Kinematica, Bohemia, NY) in a 5 ml conical tube containing 4 ml of RTL buffer (Qiagen, Toronto, ON). Based on the weight of the testes prior to freezing, an aliquot of the homogenate was collected and re-suspended in additional RLT buffer so that the amount of homogenized tissue did not surpass 30 mg/ml of RLT buffer. Entire frozen ovaries from PND 21 were processed similarly to testicular samples, except that the entire ovary was homogenized in 1 ml RLT buffer.

To ensure proper homogenization, tissue samples were further processed with a QIAshredder column (Qiagen) prior to RNA isolation with a RNeasy Mini Kit (Qiagen). RNA isolation was done as per the manufacturer’s instructions with the optional on-column gDNA elimination step. All RNA samples had RIN values greater than 9.8 measured using the Bioanalyzer 6000 (Agilent Technologies, Santa Clara, CA) and 260/280 and 260/230 ratios greater than 1.95 and 2.0 respectively, measured by NanoDrop 2000 Spectrophotometer (ThermoFisher Scientific, Wilmington, DE).

#### Blood Collection and Processing

Whole blood was collected by cardiac puncture using a 1 ml syringe and 25 G 5/8 inch needle at PND 21. Whole blood was collected in a BD Vacutainer SST tube (Becton, Dickinson and Company, Mississauga, ON) by negative pressure. The tubes were inverted several times and allowed to clot at room temperature for 30 minutes. To isolate serum, tubes were spun at 1000 × g in an Allegra-X 15 R benchtop centrifuge (Beckman Coulter, Pasadena CA) at 4 °C with a SX4750 swinging bucket rotor. Serum was aliquoted and kept at −80 °C until further use.

### Serum Gonadotropin and Hormone Levels

Serum estradiol, progesterone and testosterone levels were assessed using enzyme-linked immunosorbent assay kits (reference numbers IB79103, IB79105 and IB79106, respectively; IBL America, Minneapolis, MN) according to the manufacturer’s instructions. LH and FSH levels were assessed by the Ligand Assay & Analysis Core of the University of Virginia School of Medicine Center for Research in Reproduction using Millipore Pituitary Panel Multiplex kits (EMD Millipore, Saint Charles, MO).

### Serum Biochemistry

Analyte concentrations were determined with a Vitros 250 (Ortho-Clinical Diagnostics, Markham, ON) with dry-slide matter.

### *Ex-vivo* Organ Culture and Radioimmunoassay

Testes from PND 3 pups were collected and cut into 5–10 fragments to maintain tissue integrity during short-term culture. Fragments were placed on a 60 µm nylon mesh, which in turn was placed in a 24-well plate with a 12 mm Millicell Cell Culture Insert (Catalogue #PICM01250, EMD Millipore, Etobicoke, ON). Each well contained 300 µl of DMEM/Ham’s F12 (1:1) supplemented with GlutaMax (Gibco) and 80 µg/ml of gentamycin (basal media) (Sigma-Aldrich). Samples were incubated at 37 °C with 3.5% CO_2_. Fragments were kept at the air-liquid interface.

All testicular fragments were maintained in culture with basal media for the initial 24 hours. The left testis from each animal provided fragments for the basal condition, while the right testis fragments were stimulated with 50 ng/ml human chorionic gonadotropin (hCG) on days two and three of culture. The medium was collected to quantify testosterone with an in-house radioimmunoassay kit, as previously described^30^.

### Morphometric Analysis of Gonocytes

PND 3 testes were paraffin embedded. Serial sections of 4 µm were rehydrated and stained with H&E histological stain. Slides were digitally scanned with an Aperio AT Turbo (Leica Microsystems Inc., Concord, Ontario) at 40x magnification. To quantify the number of multinucleated gonocytes, the outlines of tubules were traced using ImageJ^61^. Only perpendicular cross-sections were quantified. To determine if a tubule was perpendicular, the major and minor axis had to be within 10% of each other. Gonocytes were considered within the focal plane if they had clearly defined cytoplasm and borders.

### Quantitative qPCR

Using the RT^2^ First Strand Kit (Qiagen) 400 ng of total RNA was cleaned-up of potential genomic DNA contamination, and converted to cDNA as per manufacturer’s instructions. A portion of cDNA was mixed thoroughly with the 2x RT2 SYBR Green Mastermix to prepare 75 reactions as per kit proportions. The mixture was pipetted into a frosted 384-well Custom RT^2^ PCR Primer Array (Supplemental Table S6, Custom Array #CLAR21524; Qiagen) using a Janus Standard Workstation (Perkin Elmer, Woodbridge, ON) equipped with Varispan Arm and 500 µL dispensing syringe. Gene transcripts were selected based on previous findings. Several steroidogenic enzymes were selected for their role in cholesterol transport (Star, Tspo, Scarb1) or in the biotransformation of steroid hormones (Cyp11a1, Hsd3b1, Cyp17a1, Hsd17b3, Cyp19a1, Srd5a1, Srd5a2). Transcripts involved in feedback mechanisms expressed in the testis and ovary (Fshr, Lhcgr), and secreted signalling molecules (Inhba, Inhbb) were quantified to assess the integrity of the hypothalamic-pituitary-gonadal axis. Many of these transcripts have been reported previously to be differentially modulated following phthalate exposure^15, 36, 62, 63^. In addition to steroidogenesis, receptors for steroid (Ar, Esr1, Esr2) or xenobiotic signalling (Ahr), transcription factors important for gonadal function and development (Kitl, Sf1, Rhox5, Nr5a2), and genes of reproductive interest modulated by phthalates^64, 65^ (Insl3, Gja1) were also assessed.

The plates were sealed and immediately run on with a Bio-Rad CFX384 (Bio-Rad, Saint-Laurent, QC). PCR was initiated by holding the temperature at 95 °C for 10 minutes. This was followed by 40 cycles of 95 °C for 15 seconds with a ramp speed of 1 °C/sec and 60 °C for 1 minute. A melt curve was generated to ensure the specificity of the PCR reaction. All targets had a single melt curve peak. Threshold cycle values were determined using CFX Manager v3.1 (Bio-Rad Laboratories, Hercules, CA) software by a baseline subtracted curve fit followed by regression analysis. All samples were pipetted as technical duplicates, and each treatment had six biological replicates. Samples with a standard deviation greater that 0.35 (or Ct difference of more than 0.5) or a Ct greater than 35 were not included in the analysis due to high variation. The housekeeping genes (*Ppia*, *Hprt*) were averaged to form a single housekeeping entity for normalization purposes. Relative quantities of each target were determined by dividing the ΔCt values for each target by the geometric mean of the control biological replicates^66^.

### Statistical Analyses

The biological replicates reported are at the level of the litter. Significance was determined by ANOVA followed by Dunnett’s post hoc test for continuous variables. For categorical variables, Fisher’s exact test was used. For qPCR data, relative quantities were log transformed prior to ANOVA followed by Dunnett’s multiple range test to account for different numerical ranges of downregulated versus upregulated genes. Outliers due to biological differences have not been removed from any of these data. Statistical calculations and visual representations were generated using GraphPad Prism 6.07 (GraphPad Software, La Jolla, CA). The data are presented without a family-wise error correction.

## Electronic supplementary material

Supplemental Information
