# Supplementary material for: In Utero and Lactational Exposure Study in Rats to Identify Replacements for Di(2-ethylhexyl) Phthalate
Source: Sci Rep. 2017 Jun 20;7:3862. doi: 10.1038/s41598-017-03979-0 (PMC5478651; doi:10.1038/s41598-017-03979-0)
Supplement: Supplementary file 1 — Supplemental Information [file 41598_2017_3979_MOESM1_ESM.pdf]

In Utero and Lactational Exposure Study in Rats to Identify Replacements for Di(2-ethylhexyl) Phthalate

SUPPLEMENTAL TABLES & FIGURES

Thomas C. Nardelli[1,+], Océane Albert[1,+], Claudia Lalancette[1], Martine Culty[1,2,4], Barbara F. Hales[1], Bernard Robaire[1,3,4*]

Departments of Pharmacology & Therapeutics[1], Medicine[2], and Obstetrics & Gynecology[3],

McGill University, Montreal, Quebec, H3G 1Y6, Canada

The Research Institute of the McGill University Health Centre[4],

Montreal, Quebec, H4A 3J1, Canada

[+]These authors contributed equally to this work.

*Corresponding author:
Bernard Robaire
Department of Pharmacology and Therapeutics
McGill University
3655 Promenade Sir William Osler, room 104
Montréal, QC Canada, H3G 1Y6
Email: bernard.robaire@mcgill.ca

Supplementary Table S1: Dam Organ Weights

	Treatment groups								
**Weights (g)**	**CORN OIL**	**DEHP30**	**DEHP300**	**DINCH30**	**DINCH300**	**BDB30**	**BDB300**	**DOS30**	**DOS300**
**Spleen**	0.19 ± 0.01	0.18 ± 0.01	0.19 ± 0.01	0.18 ± 0.01	0.18 ± 0.01	0.18 ± 0.01	0.19 ± 0.01	0.19 ± 0.01	0.20 ± 0.01
**Kidney (Paired)**	0.77 ± 0.01	0.78 ± 0.01	0.81 ± 0.02	0.79 ± 0.02	0.78 ± 0.02	0.79 ± 0.01	0.77 ± 0.01	0.75 ± 0.01	0.80 ± 0.02
**Ovaries (Paired)**	0.04 ± 0.00	0.04 ± 0.00	0.04 ± 0.00	0.04 ± 0.00	0.05 ± 0.01	0.04 ± 0.00	0.04 ± 0.00	0.04 ± 0.00	0.04 ± 0.00
**Uterus**	0.16 ± 0.01	0.16 ± 0.01	0.15 ± 0.01	0.16 ± 0.01	0.14 ± 0.02	0.12 ± 0.01	0.14 ± 0.01	0.13 ± 0.01	0.13 ± 0.01
**Lungs**	0.53 ± 0.04	0.46 ± 0.02	0.48 ± 0.02	0.47 ± 0.01	0.52 ± 0.03	0.50 ± 0.02	0.47 ± 0.02	0.50 ± 0.02	0.49 ± 0.03

Table of organ weights normalized per every 100g of body weight. None of the treatments significantly affected these organ weights in the dams necropsied at the end of lactation. Values are means ± SEM. One-way ANOVA with Dunnett's post-hoc test; n=11-16.

Supplementary Table S2: Dam Serum Parameters

Treatment groups

	CORN OIL	DEHP30	DEHP300	DINCH30	DINCH300	BDB30	BDB300	DOS30	DOS300
**Total Protein (g/L)**	59 ± 1	61 ± 2	61 ± 2	62 ± 2	59 ± 2	57 ± 2	65 ± 2	59 ± 2	62 ± 2
**Albumin (g/L)**	35 ± 1	36 ± 2	35 ± 1	36 ± 2	34 ± 2	32 ± 1	37 ± 1	34 ± 2	35 ± 1
**Albumin/Globulin Ratio**	1.4 ± 0.0	1.4 ± 0.0	1.3 ± 0.0	1.4 ± 0.1	1.4 ± 0.1	1.3 ± 0.1	1.3 ± 0.0	1.4 ± 0.0	1.3 ± 0.0
**Glucose (mmol/L)**	12.7 ± 1.1	12.6 ± 1.0	11.3 ± 0.7	12.2 ± 0.8	12.6 ± 0.8	11.6 ± 0.4	13.4 ± 1.0	11.4 ± 0.7	13.3 ± 1.1
**Blood Urea Nitrogen (mmol/L)**	9.5 ± 0.8	9.6 ± 0.4	10.2 ± 0.7	10.3 ± 0.4	8.7 ± 0.6	8.1 ± 0.8	10.3 ± 0.8	9.7 ± 0.3	9.5 ± 0.3
**Creatinine (µmol/L)**	33 ± 2	32 ± 2	33 ± 2	33 ± 2	31 ± 1	29 ± 2	33 ± 2	31 ± 2	36 ± 4
**Creatine Kinase (U/L)**	248 ± 73	142 ± 12	188 ± 56	139 ± 17	171 ± 38	796 ± 611	170 ± 29	159 ± 21	262 ± 83
**Cholesterol (mmol/L)**	2.61 ± 0.10	2.73 ± 0.13	2.30 ± 0.10	2.64 ± 0.15	2.44 ± 0.17	2.88 ± 0.22	2.81 ± 0.17	2.30 ± 0.08	2.46 ± 0.09
**Potassium (mmol/L)**	7.3 ± 0.7	6.4 ± 0.5	6.7 ± 0.4	6.5 ± 0.4	6.5 ± 0.3	6.8 ± 0.4	6.8 ± 0.5	6.1 ± 0.3	6.3 ± 0.4
**Calcium (mmol/L)**	3.05 ± 0.08	3.01 ± 0.17	3.00 ± 0.14	3.13 ± 0.16	2.86 ± 0.06	2.89 ± 0.07	3.15 ± 0.16	2.82 ± 0.04	2.92 ± 0.05
**Phosphorus (mmol/L)**	2.93 ± 0.34	2.56 ± 0.39	2.68 ± 0.17	2.67 ± 0.17	2.47 ± 0.17	3.10 ± 0.45	2.59 ± 0.18	2.14 ± 0.16	2.77 ± 0.25

None of the treatments significantly affected these serum parameters in the blood collected by cardiac puncture from dams necropsied at the end of lactation. Values are means ± SEM. One-way ANOVA with Dunnett’s post-hoc test; n=10.

Supplementary Table S3: Markers of Pup Growth at Postnatal Day (PND) 3

	Treatment groups								
	**CORN OIL**	**DEHP30**	**DEHP300**	**DINCH30**	**DINCH300**	**BDB30**	**BDB300**	**DOS30**	**DOS300**
**MALE**									
Weight (g)	8.75 ± 0.36	8.93 ± 0.27	9.12 ± 0.41	9.45 ± 0.4	8.22 ± 0.19	8.14 ± 0.28	8.31 ± 0.34	8.65 ± 0.31	8.34 ± 0.29
CRL (mm)	51.24 ± 0.69	51.44 ± 0.59	51.7 ± 0.79	52.02 ± 0.62	50.31 ± 0.28	49.94 ± 0.48	50.19 ± 0.62	50.82 ± 0.47	50.48 ± 0.53
**FEMALE**									
Weight (g)	8.45 ± 0.37	8.49 ± 0.25	8.54 ± 0.42	8.96 ± 0.36	7.72 ± 0.2	7.86 ± 0.3	7.89 ± 0.28	8.3 ± 0.31	8.05 ± 0.32
CRL (mm)	50.05 ± 0.7	50.13 ± 0.54	50.35 ± 0.87	51.14 ± 0.58	48.97 ± 0.35	48.82 ± 0.62	48.98 ± 0.54	49.81 ± 0.52	49.46 ± 0.6

Average weight and crown-rump length (CRL) of male and female pups at PND3. All pups within a litter were measured and averaged prior to averaging across litters within the same treatment (i.e. the biological replicate is the litter). No significant changes were observed in either parameter. Values are means ± SEM. One-way ANOVA with Dunnett's post-hoc test; n=11-16.

Supplementary Table S4: Organ Weights of Male and Female Pups at PND 21

	Treatment groups								
	**CORN OIL**	**DEHP30**	**DEHP300**	**DINCH30**	**DINCH300**	**BDB30**	**BDB300**	**DOS30**	**DOS300**
**MALES**									
Weight (g)	59.9 ± 1.6	59.3 ± 1.5	57.7 ± 2.7	58.5 ± 1.6	57.5 ± 1.2	52.2 ± 2.1	57.0 ± 1.6	59.1 ± 1.1	55.8 ± 2.1
Liver	4.41 ± 0.07	4.46 ± 0.12	4.36 ± 0.11	4.34 ± 0.10	4.35 ± 0.09	4.13 ± 0.10	4.43 ± 0.06	4.45 ± 0.07	4.07 ± 0.23
Spleen	0.29 ± 0.01	0.28 ± 0.02	0.30 ± 0.03	0.29 ± 0.02	0.28 ± 0.01	0.23 ± 0.02	0.27 ± 0.02	0.28 ± 0.01	0.27 ± 0.02
Kidneys(Paired)	1.25 ± 0.03	1.24 ± 0.02	1.25 ± 0.02	1.25 ± 0.02	1.23 ± 0.02	1.29 ± 0.04	1.24 ± 0.03	1.23 ± 0.02	1.27 ± 0.03
Heart	0.66 ± 0.01	0.66 ± 0.02	0.67 ± 0.02	0.69 ± 0.02	0.67 ± 0.02	0.72 ± 0.03	0.68 ± 0.04	0.66 ± 0.01	0.67 ± 0.02
Lungs(Paired)	1.32 ± 0.07	1.24 ± 0.04	1.47 ± 0.14	1.28 ± 0.0	1.397 ± 0.096	1.33 ± 0.044	1.42 ± 0.08	1.38 ± 0.07	1.30 ± 0.06
Testes (Paired)	0.26 ± 0.01	0.26 ± 0.01	0.25 ± 0.02	0.25 ± 0.01	0.25 ± 0.01	0.23 ± 0.01	0.24 ± 0.01	0.26 ± 0.01	0.24 ± 0.01
Epididymides (Paired)	0.13 ± 0.02	0.10 ± 0.01	0.10 ± 0.01	0.10 ± 0.01	0.10 ± 0.01	0.09 ± 0.01	0.10 ± 0.01	0.10 ± 0.01	0.10 ± 0.01
Seminal Vesicles(Paired)	0.04 ± 0.02	0.02 ± 0.00	0.03 ±v0.01	0.02 ± 0.00	0.02 ± 0.00	0.04 ± 0.02	0.02 ± 0.00	0.02 ± 0.00	0.02 ± 0.00
**FEMALES**									
Weight (g)	56.3 ± 1.5	57.9 ± 1.6	53.9 ± 2.4	55.8 ± 1.6	55.7 ± 1.4	53.4 ± 1.5	54.3 ± 1.5	57.2 ± 1.4	54.9 ± 2.2
Liver	4.29 ± 0.11	4.37 ± 0.09	4.3 ± 0.08	4.3 ± 0.08	4.26 ± 0.09	4.14 ± 0.08	4.35 ± 0.09	4.46 ± 0.07	4.27 ± 0.08
Spleen	0.27 ± 0.01	0.29 ± 0.01	0.27 ± 0.02	0.28 ± 0.02	0.26 ± 0.02	0.24 ± 0.02	0.26 ± 0.02	0.30 ± 0.01	0.27 ± 0.02
Kidneys(Paired)	1.26 ± 0.02	1.28 ± 0.01	1.24 ± 0.03	1.27 ± 0.03	1.24 ± 0.02	1.28 ± 0.03	1.23 ± 0.03	1.21 ± 0.02	1.31 ± 0.02
Heart	0.61 ± 0.04	0.65 ± 0.01	0.67 ± 0.02	0.66 ± 0.02	0.65 ± 0.02	0.62 ± 0.02	0.67 ± 0.03	0.62 ± 0.01	0.64 ± 0.02
Lungs(Paired)	1.13 ± 0.07	1.30 ± 0.07	1.35 ± 0.12	1.31 ± 0.06	1.37 ± 0.06	1.20 ± 0.05	1.29 ± 0.14	1.25 ± 0.09	1.27 ± 0.07
Ovaries(Paired)	1.13 ± 0.07	1.30 ± 0.07	1.35 ± 0.12	1.31 ± 0.06	1.37 ± 0.06	1.20 ± 0.05	1.29 ± 0.14	1.25 ± 0.09	1.27 ± 0.07
Uteri	0.06 ± 0.00	0.12 ± 0.05	0.07 ± 0.00	0.07 ± 0.00	0.06 ± 0.01	0.11 ± 0.05	0.06 ± 0.00	0.11 ± 0.04	0.11 ± 0.05

Table of organ weights normalized per every 100g of body weight. One pup from each litter was selected at random and necropsied. No significant changes were observed in any organ weight parameters. Values are means ± SEM. One-way ANOVA with Dunnett's post-hoc test; n=11-16.

Supplementary Table S5: Organ Weights of Male and Female Pups at PND46

Treatment groups

	CORN OIL	DEHP30	DEHP300	DINCH30	DINCH300	BDB30	BDB300	DOS30	DOS300
**MALES**									
Weight (g)	259.8 ± 5.1	255.6 ± 4.7	256.9 ± 8.3	258.9 ± 4.9	257.1 ± 5.5	238 ± 3.8	252.4 ± 6	244.9 ± 5.5	254.6 ± 5.7
Liver	5.59 ± 0.10	5.59 ± 0.11	5.50 ± 0.14	5.67 ± 0.17	5.73 ± 0.11	5.50 ± 0.12	5.72 ± 0.10	5.40 ± 0.09	5.59 ± 0.13
Spleen	0.30 ± 0.01	0.28 ± 0.01	0.31 ± 0.01	0.30 ± 0.01	0.29 ± 0.01	0.28 ± 0.01	0.31 ± 0.01	0.35 ± 0.06	0.32 ± 0.02
Kidneys	2.71 ± 0.06	2.74 ± 0.10	2.69 ± 0.08	2.75 ± 0.06	2.70 ± 0.06	2.40 ± 0.06	2.57 ± 0.10	2.37 ± 0.16	2.73 ± 0.07
Heart	0.48 ± 0.01	0.52 ± 0.03	0.51 ± 0.02	0.50 ± 0.01	0.50 ± 0.01	0.50 ± 0.01	0.50 ± 0.02	0.48 ± 0.01	0.49 ± 0.01
Lungs	0.66 ± 0.02	0.70 ± 0.03	0.72 ± 0.03	0.69 ± 0.02	0.74 ± 0.03	0.72 ± 0.03	0.78 ± 0.04	0.73 ± 0.03	0.76 ± 0.04
Testes	2.45 ± 0.06	2.40 ± 0.10	2.48 ± 0.06	2.47 ± 0.06	2.43 ± 0.05	2.43 ± 0.05	2.26 ± 0.07	2.37 ± 0.06	2.41 ± 0.06
Epididymides	0.15 ± 0.00	0.16 ± 0.01	0.15 ± 0.01	0.15 ± 0.00	0.15 ± 0.00	0.15 ± 0.01	0.15 ± 0.01	0.15 ± 0.01	0.15 ± 0.00
Seminal Vesicles	0.15 ± 0.01	0.14 ± 0.01	0.13 ± 0.01	0.15 ± 0.01	0.14 ± 0.01	0.15 ± 0.01	0.12 ± 0.01	0.16 ± 0.01	0.14 ± 0.01
**FEMALES**									
Weight (g)	187.4 ± 3.6	185.4 ± 4.3	179.4 ± 5.1	188.7 ± 2.9	181.2 ± 4.5	179.4 ± 4.4	184.9 ± 6.1	183.1 ± 3.4	181.6 ± 4.4
Liver	5.23 ± 0.11	5.12 ± 0.15	5.23 ± 0.08	5.19 ± 0.14	5.27 ± 0.09	5.01 ± 0.10	5.21 ± 0.09	5.16 ± 0.07	5.19 ± 0.09
Spleen	0.28 ± 0.01	0.25 ± 0.01	0.29 ± 0.01	0.33 ± 0.07	0.27 ± 0.01	0.27 ± 0.01	0.29 ± 0.01	0.28 ± 0.01	0.29 ± 0.01
Kidneys	1.94 ± 0.06	1.91 ± 0.05	1.86 ± 0.04	1.74 ± 0.13	1.82 ± 0.04	1.81 ± 0.06	1.90 ± 0.07	1.84 ± 0.04	1.86 ± 0.05
Heart	0.46 ± 0.01	0.46 ± 0.01	0.49 ± 0.01	0.48 ± 0.01	0.48 ± 0.01	0.48 ± 0.01	0.47 ± 0.01	0.46 ± 0.01	0.47 ± 0.01
Lungs	0.74 ± 0.04	0.72 ± 0.03	0.76 ± 0.05	0.72 ± 0.03	0.75 ± 0.03	0.69 ± 0.02	0.78 ± 0.06	0.75 ± 0.03	0.68 ± 0.04
Ovaries	0.05 ± 0.00	0.06 ± 0.01	0.06 ± 0.00	0.06 ± 0.00	0.06 ± 0.00	0.07 ± 0.01	0.06 ± 0.00	0.06 ± 0.01	0.08 ± 0.02
Uteri	0.21 ± 0.02	0.17 ± 0.02	0.19 ± 0.01	0.19 ± 0.02	0.18 ± 0.01	0.18 ± 0.02	0.22 ± 0.02	0.16 ± 0.01	0.22 ± 0.02

See footnote of previous table.

Supplementary Table S6: RT^2 Profiler Primers and Catalogue Numbers

Gene Symbol	Catalogue Number	Gene Name	Refseq Accession
**Ahr**	PPR52899F	Aryl Hydrocarbon Receptor	NM_013149
**Ar**	PPR44497A	Androgen Receptor	NM_012502
**Cyp11a1**	PPR42479A	Cytochrome P450, Family 11, Subfamily A, Polypeptide 1	NM_017286
**Cyp17a1**	PPR44710A	Cytochrome P450, Family 17, Subfamily A, Polypeptide 1	NM_012753
**Cyp19a1**	PPR47164A	Cytochrome P450, Family 19, Subfamily A, Polypeptide 1	NM_017085
**Eef2**	PPR50864A	Eukaryotic Translation Elongation Factor 2	NM_017245
**Esr1**	PPR44939B	Estrogen Receptor 1	NM_012689
**Esr2**	PPR48980A	Estrogen Receptor 2	NM_012754
**Fshr**	PPR61699B	Follicle Stimulating Hormone Receptor	NM_199237
**Gja1**	PPR44801A	Gap Junction Protein, Alpha 1	NM_012567
**Hprt1**	PPR42247F	Hypoxanthine Phosphoribosyltransferase 1	NM_012583
**Hsd17b3**	PPR45110A	Hydroxysteroid (17-Beta) Dehydrogenase 3	NM_054007
**Hsd3b1**	PPR45361B	Hydroxy-Delta-5-Steroid Dehydrogenase, 3 Beta- And Steroid Delta-Isomerase 1	NM_001007719
**Inhba**	PPR44530A	Inhibin Beta-A	NM_017128
**Inhbb**	PPR53036A	Inhibin Beta-B	NM_080771
**Insl3**	PPR50254C	Insulin-Like 3	NM_053680
**Kitlg**	PPR06678A	KIT ligand	NM_021843
**Lhcgr**	PPR45301B	Luteinizing hormone/choriogonadotropin receptor	NM_012978
**Nr5a2**	PPR49556A	Nuclear receptor subfamily 5, group A, member 2	NM_021742
**Ppih**	PPR57387B	Peptidylprolyl isomerase H	XM_001073803
**Rhox5**	PPR49786A		
**Rpl13**	PPR42351A	Ribosomal protein L13	NM_031101
**Scarb1**	PPR52707A	Scavenger receptor class B, member 1	NM_031541
**Sf1**	PPR42393A	Splicing factor 1	NM_058210
**Srd5a1**	PPR43427F	Steroid-5-alpha-reductase, alpha polypeptide 1 (3-oxo-5 alpha-steroid delta 4-dehydrogenase alpha 1)	NM_017070
**Srd5a2**	PPR65848B	Steroid-5-alpha-reductase, alpha polypeptide 2 (3-oxo-5 alpha-steroid delta 4-dehydrogenase alpha 2)	NM_022711
**Star**	PPR45414A	Steroidogenic acute regulatory protein	NM_031558
**Tspo**	PPR06787A	Translocator protein	NM_012515

Ovarian Steroidogenic Gene Expression

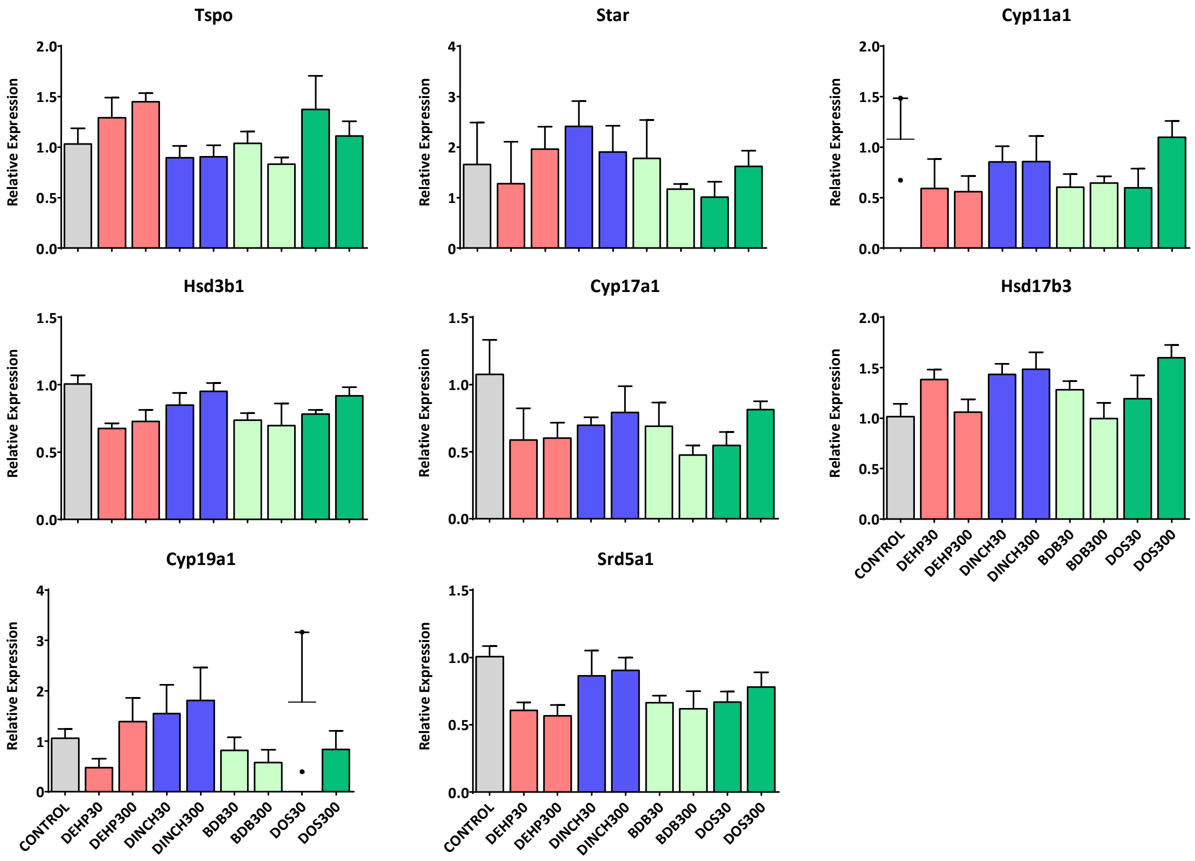

Supplemental Figure S61: Quantification of gene transcripts involved in steroidogenesis from PND 21 ovaries. The data have not been shown if a transcript was not detected or the data are too variable (SD ≥ 0.35 between replicates). A full list of primers is provided as Supplemental Table S6. Bars represent means ± SEM; n=2-6. Single points show individual replicates when n<2. One-way ANOVA, post-hoc Dunnett's multiple range test.

Ovarian Quantitative PCR Panel

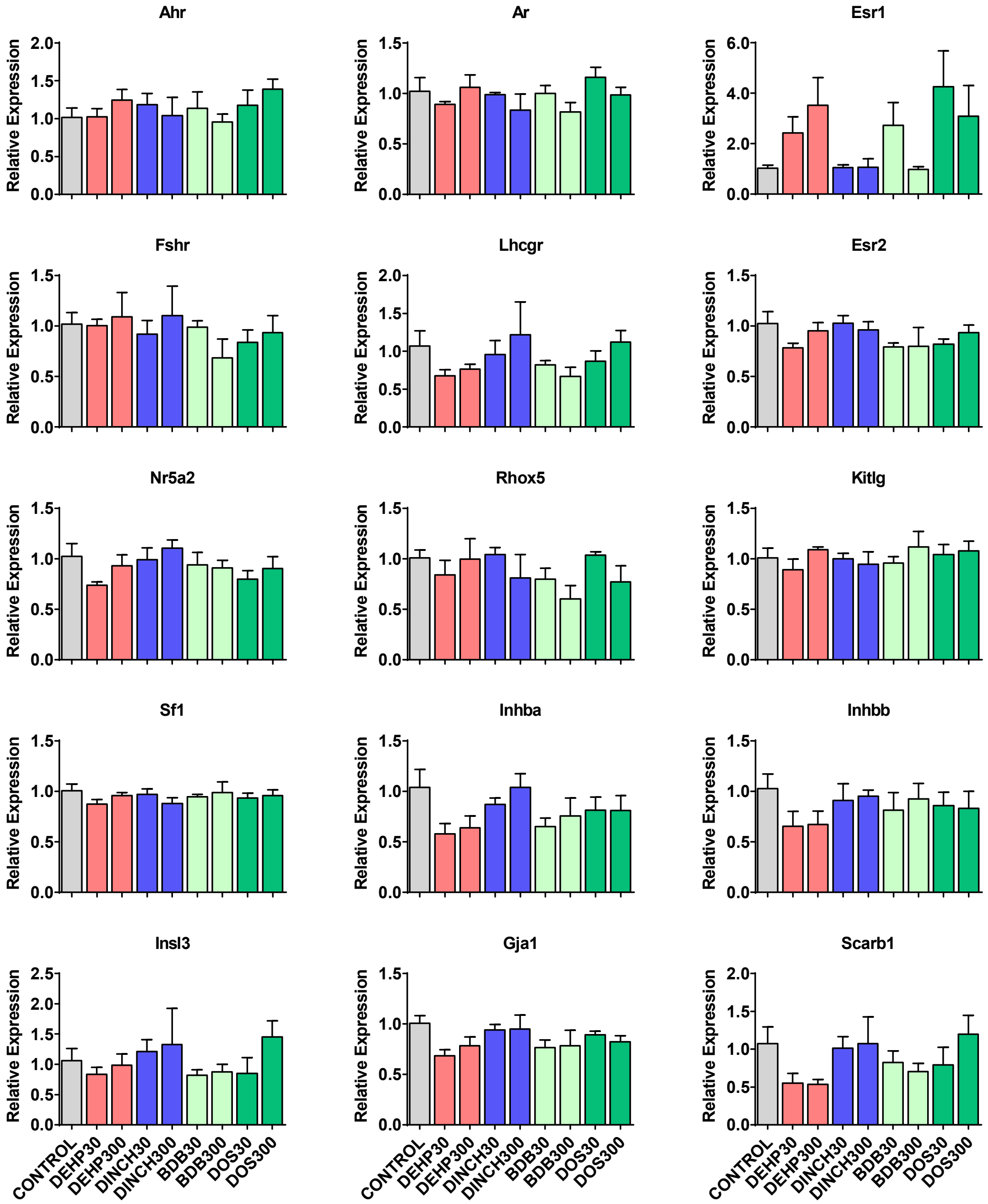

Supplemental Figure S2: qPCR array from PND 21 pup ovaries. The array includes gene transcripts that are important in reproduction and some targets that were previously reported to be affected by DEHP treatment. The data have not been shown if a transcript was not detected or the data are too variable (SD ≥ 0.35 between replicates). A full list of primers is provided as Supplemental Table S6. Bars represent means ± SEM; n=2-6. Single points show individual replicates when n<2. One-way ANOVA, post-hoc Dunnett's multiple range test.

Testicular Steroidogenic Gene Expression

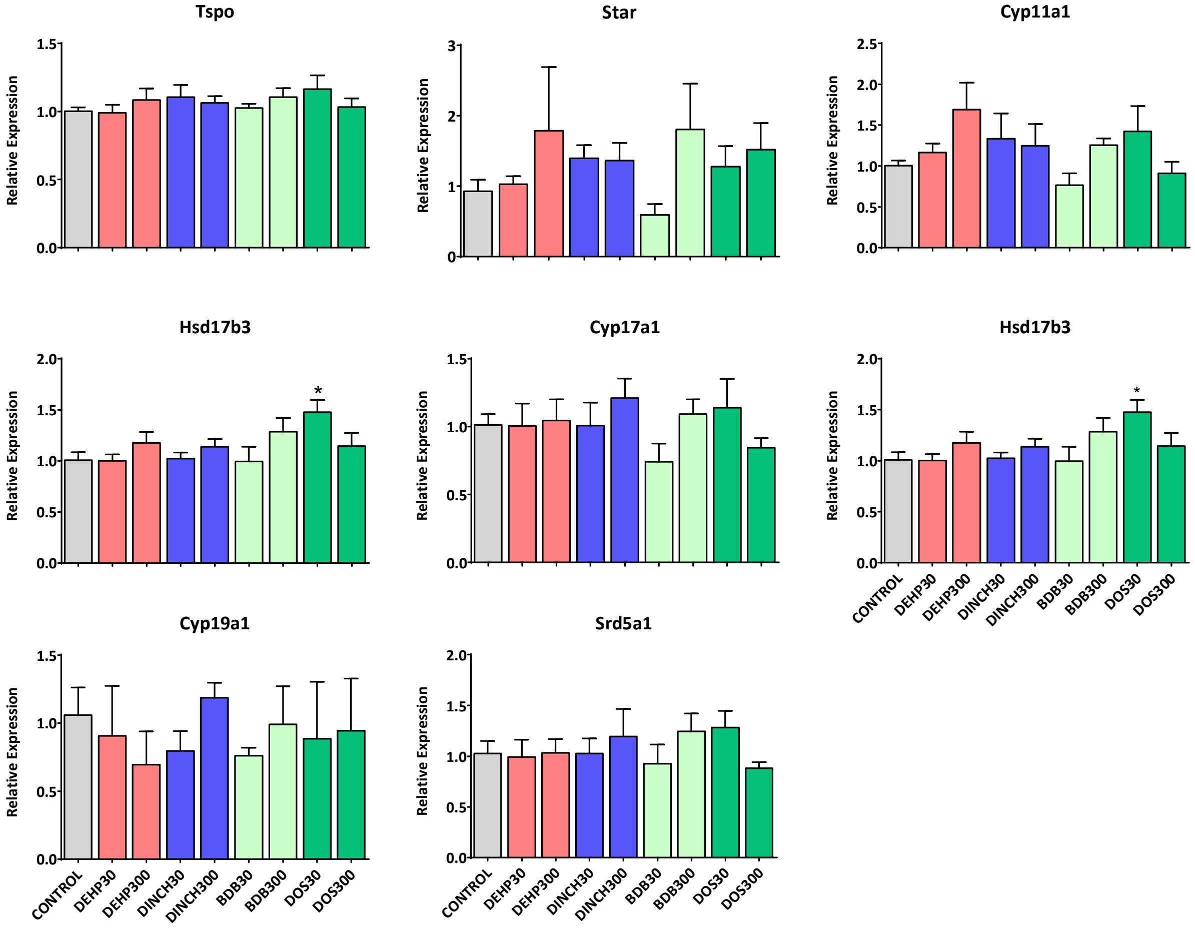

Supplemental Figure S3: Quantification of gene transcripts involved in steroidogenesis in the PND 21 testes. The data have not been shown if a transcript was not detected or the data are too variable (SD ≥ 0.35 between replicates). A full list of primers is provided as Supplemental Table S6. Bars represent means ± SEM; n=2-6. Single points show individual replicates when n<2. One-way ANOVA, post-hoc Dunnett's multiple range test.

Testicular Quantitative PCR Panel

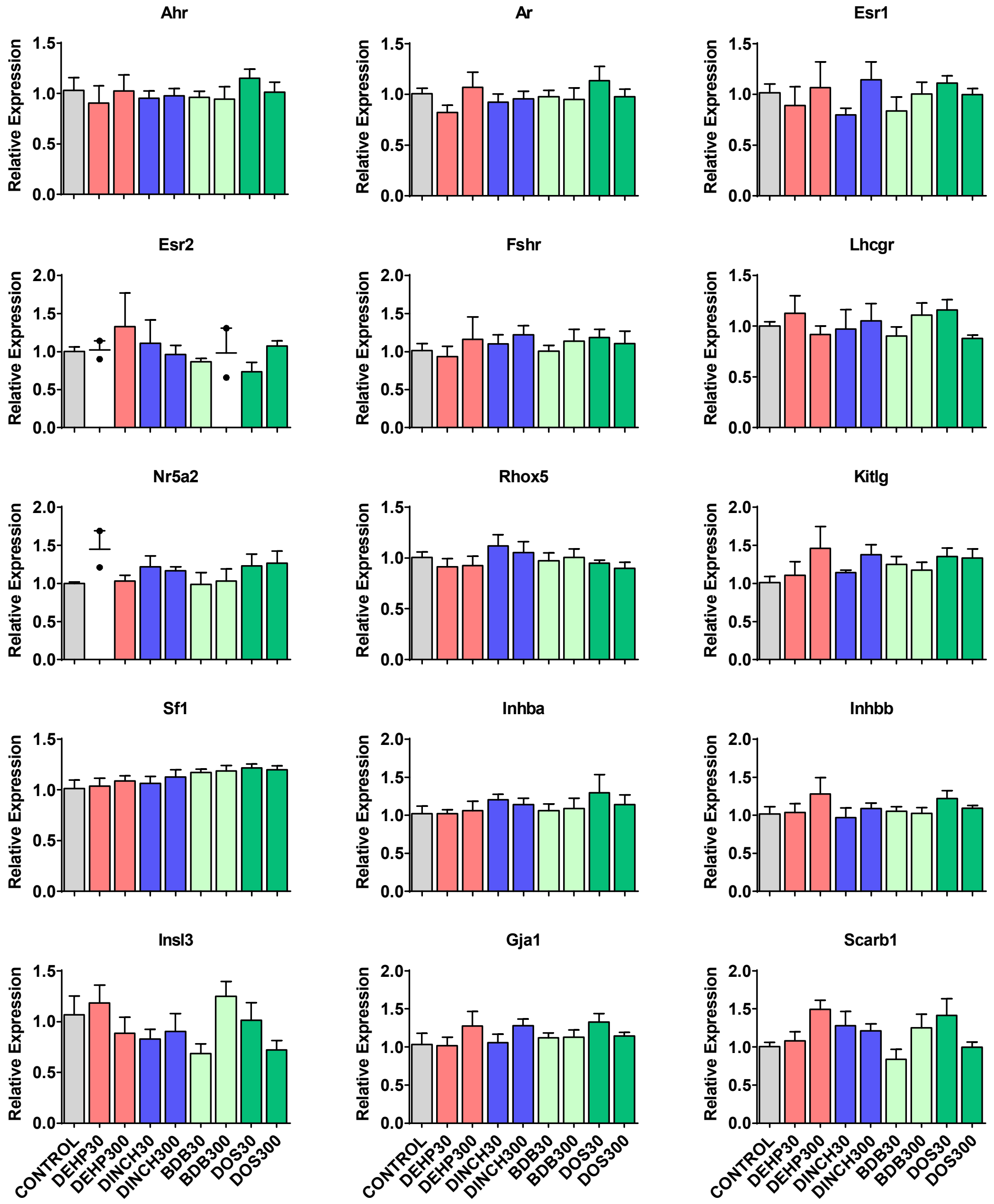

Supplemental Figure 4S: qPCR array from PND 21 pup testes. The array includes gene transcripts that are important in reproduction and some targets previously reported to be affected by DEHP treatment. The data have not been shown if a transcript was not detected or the data are too variable (SD ≥ 0.35 between replicates). A full list of primers is provided as Supplemental Table 1. Bars represent means ± SEM; n=2-6. Single points show individual replicates when n<2. One-way ANOVA, post-hoc Dunnett's multiple range test.
